# The scent gland chemistry of Gagrellinae (Opiliones, Sclerosomatidae): evidence for sequestration of myrmicacin in a species of *Prionostemma*

**DOI:** 10.1007/s00049-022-00373-9

**Published:** 2022-05-17

**Authors:** Günther Raspotnig, Michaela Bodner, Julia Blesl, Carlos Viquez

**Affiliations:** 1 Institute of Biology, University of Graz, Graz, Austria; 2 National System of Conservation Areas (SINAC), San Jose, Costa Rica

**Keywords:** Harvestmen, Chemosystematics, 3-Hydroxydecanoic acid, Chemical defense, Eupnoi, Palpatores

## Abstract

The scent gland secretion of an undetermined species of *Prionostemma* from Costa Rica was analyzed by gas chromatography–mass spectrometry and shown to consist of medium-chain carboxylic acids (mainly octanoic acid) and a *ß*-hydroxy-carboxylic acid, eventually identified as myrmicacin (= (*R*)-3-hydroxydecanoic acid). While scent gland secretions in harvestmen have traditionally been considered to be products of de novo synthesis, we here provide evidence for the unusual case of sequestration-derived scent gland constituents: at least myrmicacin appears to be sequestered from leaf-cutter ants that constitute a part of the prey of the *Prionostemma*-species herein investigated. This is the first report on the scent gland chemistry of the sclerosomatid subfamily Gagrellinae as well as on a possible sequestration mechanism in harvestmen.

## Introduction

The arachnid order Opiliones (harvestmen) is characterized by unique prosomal exocrine glands. These so-called scent glands or repugnatorial glands have been recognized as organs for defense against predators (Martens 1978; Gnaspini and Hara 2007) but may serve additional functions in microbial protection and intraspecific communication (Holmberg 1986; Machado et al. 2002; Schaider and Raspotnig 2009). Scent gland exudates are chemically megadiverse and taxon-specific, comprising naphthoquinones and methyl ketones in the Cyphophthalmi; benzoquinones, phenolics and alkaloids in the Laniatores; and benzo- and naphthoquinones along with an array of open-chain compounds in the Palpatores (= Eupnoi and Dyspnoi) (Wiemer et al. 1978; Ekpa et al. 1985; Raspotnig 2012; Raspotnig et al. 2014, 2015a, 2017).

Recent attempts to link all these compound groups within a consistent biosynthetic framework more and more paint a logical chemosystematic picture of harvestmen chemistry that reflects the evolutionary history of secretion chemistry from ancient harvestmen over million years of diversification to the richness of chemical classes and compounds in extant taxa. While recent studies focused on the evolution and taxonomic distribution of phenolics and quinones (e.g., Raspotnig et al. 2015b, 2017), the evolutionary history of other chemical classes of harvestmen scent glands has remained unclear. For instance, open-chain compounds, such as various methyl- and ethyl-ketones, vinyl-ketones, alcohols and aldehydes, represent predominant classes in the glands of Cyphophthalmi (e.g., Raspotnig et al. 2005), some Laniatores (e.g., Wouters et al. 2013), and many Palpatores (e.g., Ekpa et al. 1985). Yet, it is not clear (i) which and how many subclasses of open-chain compounds exist, (ii) how these are distributed across harvestmen taxa, (iii) whether different open-chain compounds share a common evolutionary origin, and (iv) how different open-chain compounds of various taxa may be biosynthetically linked.

One family of the Eupnoi, the Sclerosomatidae, is of particular interest as it possibly represents a lineage producing exclusively acyclic secretions (“sclerosomatid compounds” sensu Raspotnig 2012). So far, sclerosomatid gland chemistry has been shown to constitute a distinct class of related open-chain compounds, with 4,6-dimethyl-branched ethyl-ketones representing the leading structures (e.g., Meinwald et al. 1971; Jones et al. 1976, 1977).

Available data, however, are highly biased and rely on a limited number of closely related species of North American leiobunines of genera *Leiobunum* and *Hadrobunus* (Ekpa et al. 1985). The Sclerosomatidae, however, is a large family of about 1300 extant species in four subfamilies, Leiobuninae, Sclerosomatinae, Gagrellinae, and Gyinae (Kury 2013). The latter subfamily, Gyinae, is considered misplaced in sclerosomatids and was classed with Phalangiidae on the basis of genetic data (Hedin et al. 2012). Correspondingly, gyines do not produce open-chain compounds, but benzoquinones (Raspotnig et al. 2017). The scent gland chemistry of the two remaining subfamilies, Sclerosomatinae and Gagrellinae, has remained unstudied so far.

Following our long-term attempt to fully characterize the gland chemistry of Sclerosomatidae, we here report on the secretions of Gagrellinae, by focusing on an undetermined *Prionostemma*-species from Costa Rica.

## Materials and methods

**Fig. 1:**
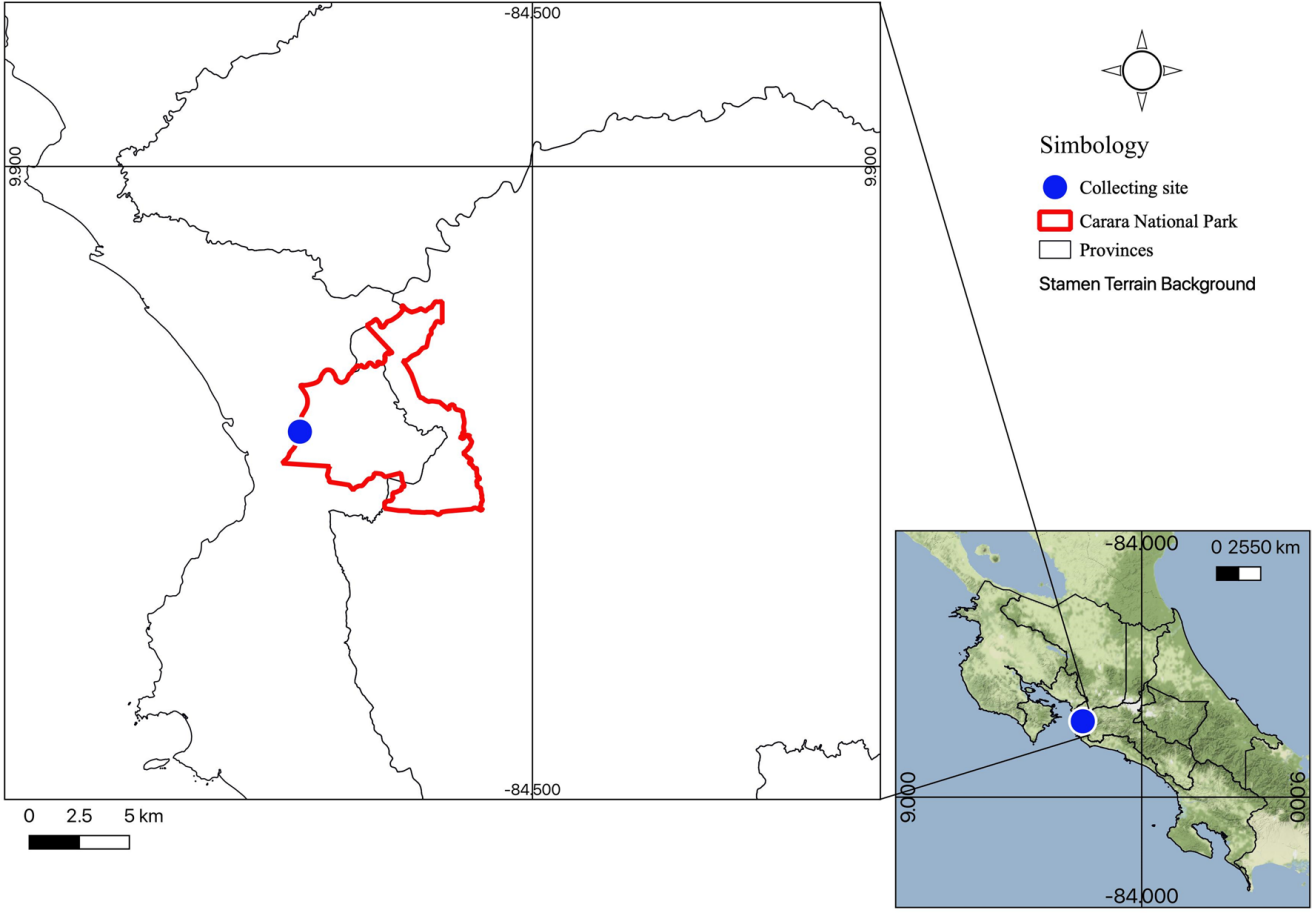
Collection site of *Prionostemma* sp. in Costa Rica

Forty individuals of *Prionostemma* sp. were collected during two collection trips in Carara National Park, Costa Rica (Fig. 1), and sent alive to the Institute of Biology of the University of Graz, Austria. Carara National Park was declared a biological reserve in 1978, later became a national park in 1998 (SINAC 2021). Located in the Puntarenas and San José provinces, Carara is a transition between tropical dry forests of the North with wet tropical forests from the South and has an area of 5242 hectares. In both expeditions, the specimens were observed during the night, between 7:00 p.m. and 12:00 p.m., always actively walking on the litter along the Araceas trail (9.77943 N; − 84.60568 W). The collections were supported under permissions R-016-2019-OT-CONAGEBIO and R-021-2019-OT-CONAGEBIO.

Scent gland secretions were extracted individually from live specimens (i.e., those that survived the transport: this was one female from the first collection; three females from the second collection). From one specimen, scent gland secretions were collected with pieces of filter paper directly after discharge from ozopores. The filter paper pieces were immediately extracted in methylene chloride, and the crude extract was used for gas chromatography-mass spectrometry (GC–MS). From the remaining three specimens individual whole-body extracts were prepared, containing quantitatively extruded scent gland secretion. In addition, 10 freshly dead individuals (5 females, 5 males) were used for a pooled extraction in methylene chloride.

Analyses were performed on two GC–MS systems (GC 2000/Voyager and Trace GC/DSQI, both from Thermo, Vienna, Austria), equipped with a 30 m ZB-5 capillary column (5%-phenyl-95%-dimethylpolysiloxane; from Phenomenex, Aschaffenburg, Germany) and a 30 m HP-chiral-20B column (*ß*-cyclodextrin in (35%-phenyl)-methylpolysiloxane; from Agilent, Vienna, Austria), respectively. MS parameters were EI at 70 eV, ion source at 200° (DSQ) and 170° (Voyager), interface at 310° (DSQ) and 245 °C (Voyager). We used the following GC-parameters and temperature programs: injector at 240 °C (both instruments); temperature program 1 (Trace GC-DSQ with ZB-5): 50 °C (1 min), with 10 °C/min to 300 °C; 5 min isotherm; temperature program 2 (GC 2000-Voyager with HP chiral-20B-column): 70 °C (1 min), with 1 °C/min to 230 °C, then 5 min isotherm.

Reference compounds and compounds for synthesis such as octanal, ethyl-2-bromoacetate, octanoic-, nonanoic-, decanoic acid, racemic 2-hydroxydecanoic acid as well as enantiopure *(R)*-3-hydroxydecanoic acid were purchased from Sigma-Aldrich, Vienna, Austria. Racemic 3-hydroxydecanoic acid was synthesized following a modified protocol of Sailer et al. (2015), via Reformatsky reaction from octanal and ethyl-2-bromoacetate to prepare ethyl 3-hydroxydecanoate and subsequent saponification with aqueous NaOH to yield a 1:1-mixture of *R*- and *S*-isomers of 3-hydroxydecanoic acid. To correctly assign the stereoisomers, the mixture was chromatographed on a chiral phase (see above), and compared to authentic *(R)*-3-hydroxydecanoic acid. For the preparation of trimethylsilylesters/-ethers from free carboxylic acids and hydroxy acids, we used MSTFA (*N*-methyl-*N*-(trimethylsilyl)-trifluoro-acetamide in pyridine 2:1 containing 1% trimethylchlorosilane) (from Sigma-Aldrich, Vienna). Retention indices of compounds (RIs) were calculated according to Van den Dool and Kratz (1963) using an alkane standard (C_7_–C_36_).

## Results

**Fig. 2:**
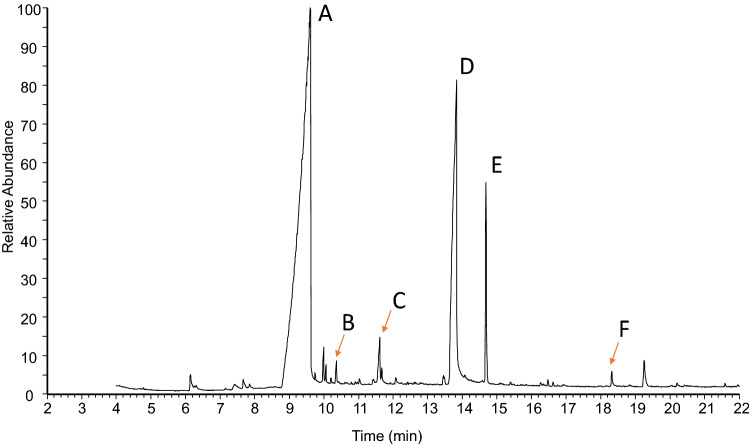
Gas chromatographic profile of the scent gland secretion directly sampled from the ozopores of a female specimen of *Prionostemma* sp.; untreated (non-derivatized) extract. Compounds: Peak **A** (octanoic acid), **B** (nonanoic acid), **C** (decanoic acid), **D** ((*R*)-3-hydroxydecanoic acid = myrmicacin), **E** (artifact arising from 3-hydroxydecanoic acid in the hot injector of the gas chromatograph), F (palmitic acid)

Secretion directly collected from ozopores as well as from whole body extracts showed medium-chain carboxylic acids (Compounds A–D in Fig. 2), as indicated by the characteristic EI-mass spectra of the compounds. There were two major compounds A and D (ratio 3:1) in the gas chromatograms, three minor or trace compounds (B, C, F), and a compound E of variable abundance. The major compound A was octanoic acid (M^+^ at *m/z* 144; > 75% of the secretion). Minor compounds B, C and F (M^+^ at *m/z* 158, 172, and 256, respectively; each compound about 1% of the secretion) were identified as nonanoic-, decanoic-, and palmitic acids. All identifications rely on GC–MS data of both derivatized and non-derivatized extracts, and comparisons to authentic standards.

**Fig. 3:**
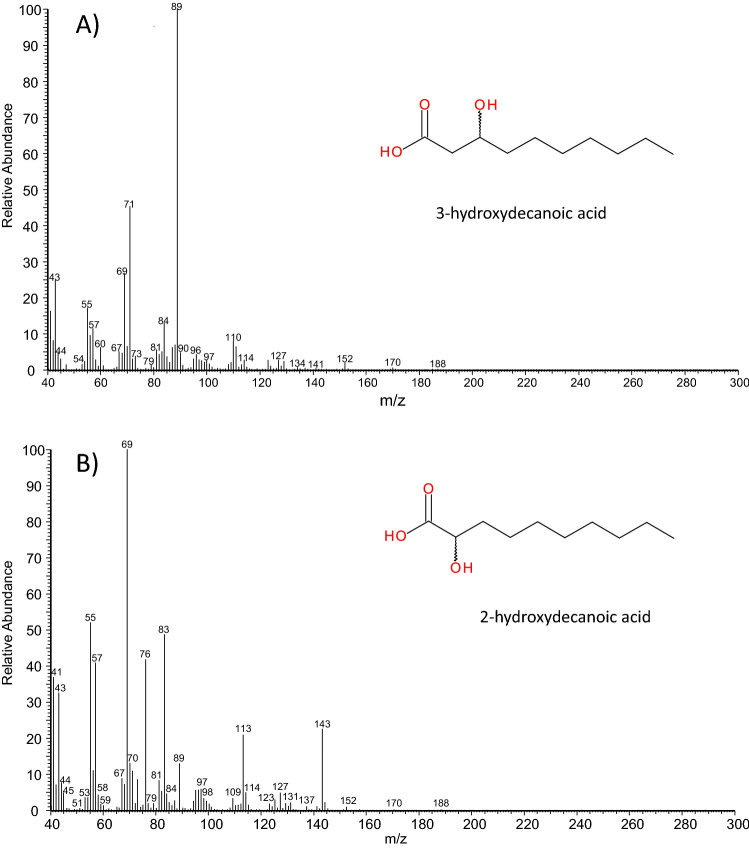
**A** Electron impact mass spectrum of 3-hydroxydecanoic acid. The major fragment (base ion at *m/z* 89) arises from α-cleavage at C_3_ of the molecule. The molecule ion is weak (at *m/z* 188). The ion from McLafferty-rearrangement (at *m/z* 60) is of only moderate intensity. **B** Electron impact mass spectrum of 2-hydroxydecanoic acid. The major fragment (base ion at *m/z* 69) is an alkene-fragment; fragments at *m/z* 143 and *m/z* 113 arise from cleavage at C_2_; *m/z* 76 is the McLafferty-fragment

**Fig. 4:**
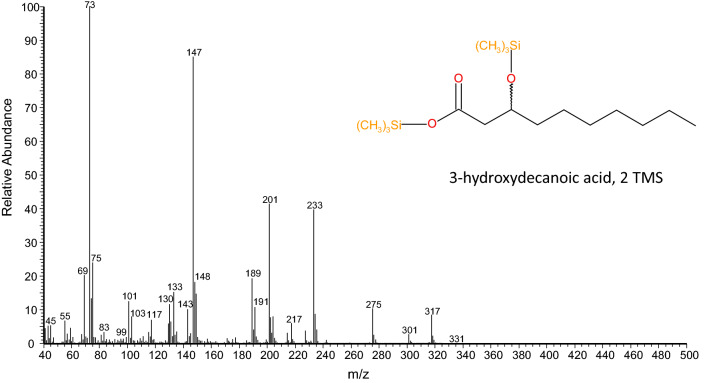
Electron impact mass spectrum of 3-hydroxydecanoic acid, 2TMS. One trimethylsilyl-group binds to the acid moiety of the molecule (forming an ester), a second to the hydroxy group (forming an ether). The molecular ion is at *m/z* 332 (188 + 72 + 72). Diagnostic fragments can be seen at *m/z* 317 (M^+^—CH_3_) as well as at *m/z* 233 and *m/z* 201 (α-cleavage at both sides of C_3_). The fragments at *m/z* 73 and *m/z* 75 correspond to the trimethylsilyl-ion and to the dimethylsilanol-ion, respectively

By contrast, compound D (about 25% of the secretion) exhibited the mass spectrum of a *ß*-hydroxycarboxylic acid, with a weak molecular ion at *m/z* 188 and a base peak at *m/z* 89 (Fig. 3A). Best hits from a library search (NIST 05) were 2-hydroxydecanoic acid and 3-hydroxydecanoic acid. A comparison to authentic reference standards showed a completely different mass spectrum for 2-hydroxydecanoic acid (base ion at *m/z* 69; Fig. 3B) and different chromatographic retention (RI_2OH-C10acid_ = 1555 vs. RI = 1546_compound D_). On the other hand, both mass spectrum and gas chromatographic retention of compound D fully corresponded to 3-hydroxydecanoic acid (RI = 1546_3OH-C10acid_). This finding was eventually confirmed by a comparison of the corresponding trimethylsilyl-derivatives (RI_3OH-C10acid-TMS_ = 1663; RI _compound D-TMS_ = 1663; EI mass spectrum in Fig. 4).

**Fig. 5:**
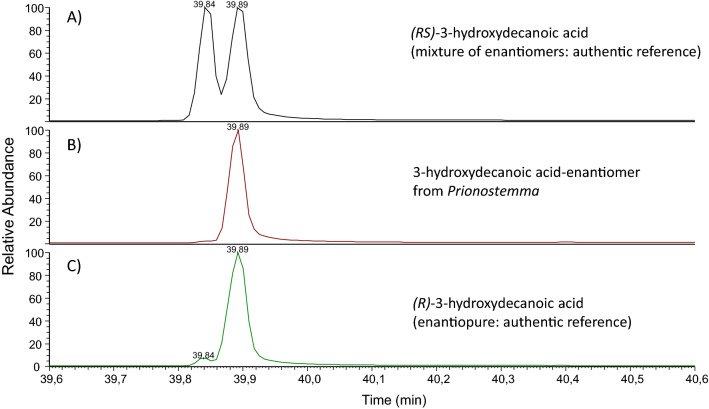
Determination of the absolute configuration of 3-hydroxydecanoic acid in the *Prionostemma*-secretion. **A** Separation of the two enantiomers in a racemic mix of synthetic 3-hydroxydecanoic acid. **B** Comparison to the enantiomer present in the *Prionostemma*-secretion. **C** Comparison to an authentic reference of enantiopure (*R*)-3-hydroxydecanoic acid

3-Hydroxydecanoic acid is a chiral compound (asymmetric C_3_), and two enantiomers exist, (*R*)-3- and (*S*)-3-hydroxydecanoic acid. To determine the absolute configuration of our compound D, we used a chiral gas chromatographic phase, demonstrating that compound D was enantiopure (*R*)-3-hydroxydecanoic acid (Fig. 5), also called myrmicacin in the literature.

Compound E appeared to be an artifact arising from 3-hydroxydecanoic acid. It was found in all samples, also in samples of reference compounds, i.e., in those containing authentic and pure hydroxydecanoic acids only. A similar artifact also occurred in samples of 2-hydroxydecanoic acid. Compound E varied in abundance with the temperature of the injector, and is considered a pyrolysis product of 3-hydroxydecanoic acid.

## Discussion

This study is intended a step towards a complete characterization of “sclerosomatid compounds”, and we here present initial chemical data for Sclerosomatidae apart from leiobunines. We currently do not want to imply that the chemical composition of the secretion of the *Prionostemma*-species investigated here is characteristic for Gagrellinae as a whole. It may, however, be seen as an example for gagrelline secretion chemistry.

First, we report on an incidence of a very unusually composed scent gland secretion, i.e. exclusively consisting of organic acids which is so far unique for harvestmen. Though acids are not completely absent from the secretions of some other harvestmen species, they are not common at all, at best representing secretion by-products in a few species (Raspotnig, personal observation). Octanoic acid, for instance, while being a frequently detected exocrine compound in both arthropod (e.g., Schmidt et al. 2000; Attygalle et al. 2004; Raspotnig and Leis 2009) and vertebrate exudates (e.g., Waterhouse et al. 2001), has been found in a single harvestmen species only (Raspotnig et al. 2015a). Moreover, this latter species, the phalangiid *Rilaena triangularis*, shows mainly non-acid compounds in its secretion. However, some harvestmen, as recently reported for *Egaenus convexus* (again a phalangiid), may produce acid-derivatives such as lactones (Raspotnig et al. 2020).

Second, one of the acids of the *Prionostemma*-secretion has been identified as myrmicacin (= (*R*)-3-hydroxydecanoic acid). Myrmicacin is best known for the secretions of metapleural glands in leaf-cutter ants (Maschwitz et al. 1970; Do Nascimento et al. 1996; Ortius-Lechner et al. 2000; Vieira et al. 2012) where it has originally been detected (Schildknecht and Koob 1971). There are not many reports on the natural occurrence of this compound. Apart from leaf-cutters of genera *Atta* and *Acromyrmex*, myrmicacin has sporadically been found in the metapleural secretions of non-leaf-cutter myrmicines such as *Messor* and *Myrmica* (Schildknecht and Koob 1971; Viera et al. 2012), the pygidial glands of water beetles of genus *Laccophilus* (Dettner 1985) as well as from some microorganisms (e.g., Sjögren et al. 2003) and in royal jelly (Kodai et al. 2011). It thus came as a surprise to us to detect myrmicacin in an arachnid. Schildknecht and Knoob (1971) called myrmicacin “the first insect-derived herbicide” since it was able to inhibit the growth of germinating plant pollen in ant colonies by blocking cellular mitosis. While other antimitotic substances are not effective at later phases of mitosis, myrmicacin was found to inhibit mitotic progression at all stages, including metaphase and anaphase (e.g., Iwanami 1978).

**Fig. 6:**
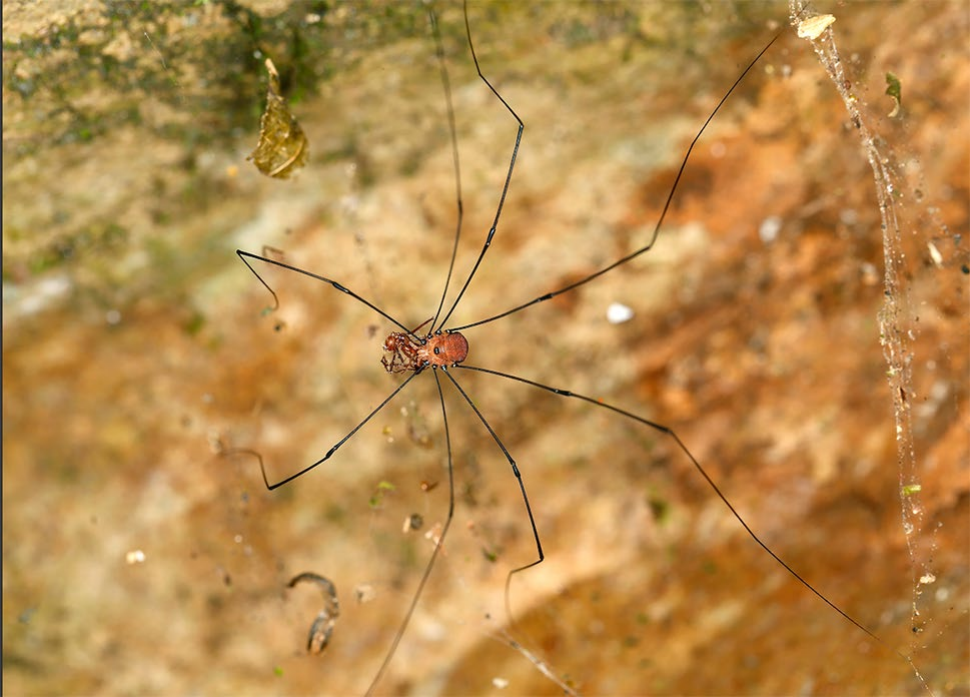
An individual of *Prionostemma* sp. is feeding on a leaf-cutter ant (photograph by Carlos Viquez)

Third, there is evidence that myrmicacin is not produced by individuals of *Prionostemma* themselves but sequestered from nutritional sources: We indeed observed *Prionostemma*-individuals feeding on leaf-cutter ants that might represent the original source of myrmicacin in *Prionostemma* (Fig. 6). Consequently, we cannot rule out the possibility that the other compounds of the secretion—all of which are medium-chain carboxylic acids, chemically close to myrmicacin—are sequestered either. Sequestration as a mechanism to build-up scent gland compounds appears to be a hitherto unique case in harvestmen that, with the tacit understanding, have generally been considered to produce scent gland components by de novo synthesis. There are mainly indirect arguments for a de novo synthesis of scent gland secretion constituents in harvestmen: one is certainly the production of taxon-specific secretions, i.e., related species produce the same or biochemically related components, irrespective of geographical location and ecological conditions, making the hypothesis of shared biosynthetic pathways to similar components/component classes very plausible. Such a de-novo synthesis also represents the backbone of the theoretical basis for harvestmen phylogenetic chemosystematics (e.g., Raspotnig et al. 2015b, 2017). On the other hand, a definite proof for the de novo synthesis of scent gland secretions is missing for most taxa. Only for phenolics and benzoquinones from laniatoreans, de novo synthesis is likely, as evidentiary shown by Rocha et al. (2013) who used labelled acetate and propionate in feeding experiments. A possible contribution of symbiotic bacteria has though not been explicitly excluded.

Scent gland secretions in harvestmen are considered to be mainly for defense (e.g., Martens 1978; Gnaspini and Hara 2007), and with respect to the acids found in the present study—irrespective of their origin—this is most likely true for *Prionostemma* as well. There is a number of arthropods that use acids for effective predator repellence, including the formic acid-producing glands of formicine ants and certain beetles as well as the opisthosomal, mainly acetic acid-producing glands of whip scorpions (Eisner 1962; Schmidt et al. 2000; Haupt and Müller 2004). Other arachnids, such as certain oribatid mites may also produce acids but use these compounds to build up water-repellent layers on their cuticle (Raspotnig and Leis 2009; Brückner et al. 2015). The mixture of myrmicacin and some medium-chain n-alkanoic acids in *Prionostemma* is also functionally of interest, probably combining predator defense with antimicrobial protection: regarding the mitosis-inhibiting effect of myrmicacin and its inherent antibacterial and antifungal properties (e.g., Iizuka et al. 1979; Iwanami et al. 1979), the compound may serve as a protection against the proliferation of microorganisms at the outer surface of the harvestmen’s cuticle. The large amount of octanoic acid, however, indicates a primarily defensive function of the secretion, but octanoic acid and other short- to medium-chain carboxylic acids are also known to reduce microbial growth (“myrmic acids”: Iwanami and Iwadare (1979)).

Acids from the *Prionostemma*-secretion add a novel class of open-chain compounds to the scent gland chemistry of Opiliones, but also complicate harvestmen chemosystematics. The possibility of sequestration of compounds may blur the chemosystematic picture of harvestmen scent glands that aims to reflect a biosynthetically linked and evolutionary-based tree of chemical classes/compounds that diversified during evolution. On the other hand, the possibility of sequestration of compounds adds a highly interesting aspect to chemosystematic research that has yet poorly been addressed: as in poison frogs, that sequester toxic alkaloids from their arthropod prey and though show specific patterns of alkaloids in their skin (e.g., Daly et al. 1994; Saporito et al. 2009), the mechanisms behind sequestration still underlie evolution, producing chemosystematic specificity on a next level. We know that the sequestration of compounds, mainly from plant sources, as well as the use of sequestered compounds in defensive/toxic secretions is quite common in several groups of arthropods, such as Lepidoptera and Coleoptera (e.g., Duffey 1980; Nishida 1994; Boland 2015; Petschenka and Agrawal 2016), but we still consider it an exception in harvestmen.
